# Educational workshops on telenursing in the monitoring of chronic conditions: an experience report

**DOI:** 10.1590/0034-7167-2025-0246

**Published:** 2026-07-06

**Authors:** Iven Giovanna Trindade Lino, Luciana de Alcantara Nogueira, Elen Ferraz Teston, Sonia Silva Marcon

**Affiliations:** I Universidade Estadual de Maringá. Maringá, Paraná, Brazil; II Universidade Federal do Paraná. Curitiba, Paraná, Brazil; III Universidade Federal de Mato Grosso do Sul. Campo Grande, Mato Grosso do Sul, Brazil

**Keywords:** Primary Health Care, Nursing, Telenursing, Chronic Disease, Information and Communication Technologies., Atención Primaria de Salud, Enfermería, Teleenfermería, Enfermedad Crónica, Tecnologías de la Información y las Comunicaciones.

## Abstract

**Objectives:**

to report on educational workshops on telenursing use for monitoring individuals with hypertension and diabetes in Primary Health Care.

**Methods:**

this experience report, conducted between May and July 2021, involved a survey of the characteristics of the care provided, a discussion of possibilities of telenursing, instrument development, and technology testing.

**Results:**

a situational assessment revealed dissatisfaction with the outcomes of the care provided. The 16 participating nurses recognized telenursing’s potential, but highlighted the lack of time and insufficient human resources and infrastructure as potential limitations. They participated in the development of a form for assessing patients’ suitability and risk stratification, as well as a nursing consultation script and care plan.

**Final Considerations:**

educational workshops are effective in training nurses, confirming the importance of continuing education to support the implementation of changes in care practice, such as telenursing use.

## RESUMEN

**Objetivos:**

informar sobre talleres educativos sobre el uso de teleenfermería para monitorear a personas con hipertensión y diabetes en Atención Primaria de Salud.

**Métodos:**

informe de experiencia, realizado entre mayo y julio de 2021, involucrando una encuesta sobre las características de la atención prestada, un análisis de las posibilidades de teleenfermería, el desarrollo de instrumentos y la prueba de la tecnología.

**Resultados:**

evaluación situacional reveló insatisfacción con los resultados de la atención prestada. Las 16 enfermeras participantes reconocieron el potencial de teleenfermería, pero destacaron la falta de tiempo y la insuficiencia de recursos humanos e infraestructura como posibles limitaciones. Participaron en el desarrollo de un formulario para evaluar la idoneidad del paciente y la estratificación del riesgo, así como de un guion de consulta de enfermería y un plan de cuidados.

**Consideraciones Finales:**

talleres educativos son eficaces para la formación de enfermeras, lo que confirma la importancia de la formación continua para apoyar la implementación de cambios en la práctica asistencial, como el uso de teleenfermería.

## INTRODUCTION

Telenursing has established itself as an innovative and effective strategy for monitoring people with chronic noncommunicable diseases, especially hypertension (HT) and diabetes *mellitus* (DM). By integrating digital technologies and remote communication, this care modality allows for increased access to healthcare services, reduced complications, and promoted self-care^(1)^.

COFEN Resolution 696/2022, amended by Resolutions 707/2022 and 717/2023, regulates nursing practices in digital health and the use of information and communication technologies (ICTs). It establishes that, in ICT-mediated actions, the consent of patients involved or their legal guardian is essential, and consent may be withdrawn at any time, resulting in consent withdrawal. In practice, the resolution authorizes nurses to use telenursing, i.e., to perform nursing consultations, interconsultations, consulting, monitoring, health education, and responding to spontaneous requests mediated by ICTs.

This care modality expands access to healthcare services in hard-to-reach areas, reduces costs, and addresses changing epidemiological profiles, such as population aging, the rise in chronic diseases, and infectious diseases. It is estimated that by 2030, more than half of healthcare services will be offered virtually, reflecting patients’ growing preference for this care model^(2)^. Furthermore, it presents itself as an essential support tool for a more dynamic and effective relationship between patients and professionals, especially in the context of Primary Health Care (PHC)^(3)^.

A study conducted with PHC nurses concluded that telenursing is considered a useful tool for monitoring people with chronic conditions, as long as adequate human, material and training resources are available^(4)^. However, it is important to emphasize that, in order to incorporate this care modality into daily work, acceptance by professionals and patients is essential, as is the existence of systematized protocols and flows that subsidize the conduct of the work, supporting health actions and the achievement of the established objectives^(5)^. It is noteworthy that research exploring patients’ and professionals’ experience with the use of telenursing is limited and conducted mostly in developed countries, such as the United Kingdom and the United States of America^(1)^_._

Given this scenario, health education can be an important strategy for improving the use of telenursing in PHC. Among the different approaches to health education, educational workshops stand out for providing a collaborative space for the exchange of ideas, enabling the analysis of challenges that require change and strengthening the connection between knowledge and practice, in addition to encouraging dialogue and the collective construction of knowledge. In short, this approach prepares professionals to identify health needs and act more effectively^(6)^.

Therefore, educational workshops focused on telenursing can raise awareness and bring professionals closer to this new care possibility. However, no studies were found in the national literature that supported nursing professionals in the use of technology in their daily care, which motivated this report.

## OBJECTIVES

To report on educational workshops on telenursing use for monitoring individuals with HT and DM in PHC.

## METHODS

This is a report of the experience lived during a matrix study developed within the 15^th^ Health Region (HR) of the state of Paraná, Brazil, carried out between May and June 2022, which proposed the use of telemonitoring as a resource to support self-management of the disease by people with DM and HT.

Participants in the experiment were 16 nurses working in Basic Health Units (BHUs) in municipalities within the 15^th^ HR. Initially, to facilitate nurse participation, the continuing health education (CHE) sector of the HR sent a letter to the PHC coordinators of the 29 municipalities informing them about the research and its objectives. They requested that the letter be forwarded to PHC nurses, along with the link to access the questionnaire on Google Forms^®^, which should be completed within 15 days. After 30 days, and given the low response rate, a new letter was sent to the coordinators reinforcing the importance of collaboration in the research.

The questionnaire was prepared by the authors and consisted of four parts: I) Sociodemographic characterization: sex, age, time since graduation and specialization in the area of family health or public health; II) Characteristics of care for people with HT and/or DM, addressing the monitoring carried out and perceptions about the advantages and disadvantages of this monitoring; III) Knowledge and perceptions about information and communication technologies and previous training; IV) Possibility of using telenursing in daily life: willingness to use it and availability of physical, structural, and human resources at the health unit, as well as interest in participating in workshops on the use of this technology. It included the Informed Consent Form, acceptance of which was a necessary condition for accessing the research form.

Nurses who expressed interest in participating in the workshops were contacted and informed about how and when they would be held. Once their interest was confirmed, they were all added to a group on the WhatsApp^®^ messaging app to facilitate communication, share information about the workshops, and strengthen their relationship with the researchers. The first meeting was scheduled according to the availability of most participants.

The workshops, a total of five, were held weekly at a time and day most participants considered most appropriate (Wednesday at 3 p.m.). All workshops were videotaped with participant permission and lasted an average of 60 minutes. The day before each meeting, the researcher sent a reminder in the WhatsApp^®^ group reinforcing the importance of everyone’s attendance and completion of the previous week’s assignment. The workshops were led by one of the authors, a nurse and doctoral student in nursing with experience in qualitative data collection and analysis who had no connection with participants.

**Figure 1:**
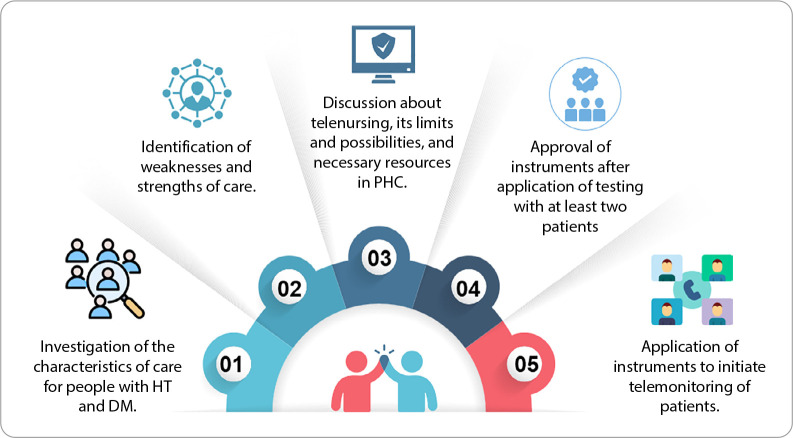
Explanatory flowchart of meeting moments

To conduct and carry out the educational workshops, after discussion and agreement among participants, an activity plan was drawn up based on the responses to the online questionnaire, which was organized into five stages, as can be seen in Figure 1.

The development of this research followed all ethical precepts regulated by Resolutions 466/2012 and 510/2016 of the Brazilian National Health Council, and the guidelines for procedures in research with any stage in a virtual environment - Brazilian National Research Ethics Commission/2021. The project was submitted for consideration and approved by the Research Ethics Committee.

## RESULTS

Of the 289 nurses working in the 15^th^ HR of Paraná, 65 responded to the questionnaire; 42 expressed interest in participating in the educational workshops; and 16 effectively participated.

In the first workshop, the group discussed the information obtained from the online questionnaire and reflected on the reality experienced by each member, highlighting the perceived weaknesses and strengths in the care provided to people with HT and/or DM at the BHU where they work. This activity aimed to know participants, understand their work context, and identify how care was provided to people with HT and/or DM.

In the second and third workshops, the principles, limitations, possibilities, and resources required for telenursing in PHC were discussed. During the discussions, concerns arose about which patients would be eligible for this type of care and the factors to be considered, such as frequency of visits to the BHU, social vulnerability, and ability to use mobile devices, as patients would need to have a phone and the ability to use technology independently.

During these two meetings, the group concluded that telenursing would be a promising strategy with the potential to contribute to the organization of care, defining the necessary steps for its operationalization. These steps are: 1) Identify patients with HT and/or DM eligible for telecontacts; 2) Use risk stratification to determine the frequency of telecontacts; 3) Conduct in-person nursing consultations to identify problems, develop a care plan, and jointly define agreed-upon goals; 4) Conduct telecontacts to monitor compliance with agreed-upon goals and assess the strengths and weaknesses faced by patients in managing their health condition; and 5) Establish new goals or return to the initial goal. However, to incorporate telenursing into the work routine, its feasibility would need to be tested and specific instruments would need to be developed to guide actions.

The group listed important aspects to include in the instruments and emphasized the need to consider the characteristics and specificities of the patients being assisted and the different realities of the municipalities, ensuring that the instruments were useful and accessible to all participants. The first instrument developed addressed patients’ suitability for telenursing and risk stratification to determine the frequency of calls. It is noteworthy that, although the Paraná Hypertension and Diabetes Guideline presents criteria for identifying patients’ risk strata, some nurses reported that their municipalities did not have a quota for certain laboratory tests required for this stratification. For this reason, these tests were not performed, much less with the required frequency, or only when a patient could afford them.

To streamline the process, based on the group’s suggestions, the researchers developed a draft instrument for further refinement. This was distributed to everyone during the week to streamline the workshop discussions. To ensure the proposed criteria could be adopted by all municipalities, they adapted the Paraná Guideline criteria, assigning points to certain conditions/situations. Thus, when assessing patient suitability, factors such as ownership of a landline or mobile phone, ability to operate a telephone and receive calls, living with others, hearing and/or visual impairments, and willingness to receive telenursing support were considered. To stratify risk, the following variables were listed: age; Body Mass Index; waist circumference; smoking; emergency room visits and/or hospitalization in the last 12 months due to uncontrolled blood pressure or blood glucose levels; surgery; complications; use of routine medications; and blood pressure and blood glucose parameters. Therefore, based on the parameters established in Paraná Guidelines, patients presenting the listed variables were considered to be at low, moderate, and high risk, as illustrated in Chart 1.

**Chart 1:** Criteria for risk stratification defined by nurses based on the Paraná Guideline, Maringá, Paraná, Brazil, 2025

Chronic condition	Variables	Risk
DM	Diagnosis of DM; blood glucose up to 130 mg/dL; uses a type of medication; follows a diet and/or practices physical activity; does not smoke; does not drink alcohol; postprandial blood glucose <180 mg/dL; absence of complications of T2DM; sought health unit in the last 12 months due to lack of glycemic control.	Low
	Uses two or more antidiabetic medications; does not follow a diet and/or does not engage in physical activity; drinks alcohol and/or smokes; postprandial blood glucose ≥ 180 mg/dL; absence of complications of T2DM; need to seek healthcare services once or twice in the last 12 months due to uncontrolled blood glucose.	Medium
	T1DM or T2DM; uses insulin; complications (macro or microvascular); sought healthcare services ≥ three times in one year due to glycemic decompensation.	High
HT	Diagnosis of HT; regular use of medication; systolic pressure of 130-139 mmHg or diastolic of 85-89 mmHg; did not seek healthcare services in the last 12 months due to uncontrolled blood pressure.	Low
	Blood pressure ≤ 130/80 mmHg; uses two to three antihypertensive medications; follows a diet and/or practices physical activity; smokes and/or drinks; sought healthcare services once or twice a year due to uncontrolled blood pressure.	Medium
	Uses two to three medications; blood pressure ≥ 180 mmHg or diastolic ≥ 110 mmHg; does not follow diet and/or physical activity; smokes and/or drinks; has cardiovascular complications; sought healthcare services more than three times in 12 months due to uncontrolled blood pressure.	High

*HT - hypertension; DM - diabetes mellitus.*

*A priori*, the group determined that risk stratification would determine the frequency of calls. For patients with lowand medium-risk HT and/or DM, whose agreed-upon goals are aimed at changing risk behaviors, calls should occur every three months, totaling four calls in a 12-month period. For patients stratified as high-risk, whose agreement focuses on case management, calls should be monthly.

However, it was considered that the established frequency could vary according to the goals agreed upon in the care plans and the evolution of patients’ health condition/behavior. In other words, in an in-person nursing consultation, nurses might encounter a patient with T1DM who, according to established criteria, would be stratified as high-risk. However, upon determining that a patient has healthy habits, satisfactory self-care, and glycemic levels within normal ranges, telecontacts would not need to be monthly, but rather quarterly.

On the other hand, patients classified as low or medium risk could exhibit significantly detrimental health behaviors, with many unhealthy lifestyle habits. In these cases, goals need to be gradually agreed upon, and some more urgent goals need to be monitored more frequently and more closely. Therefore, patients would need to be contacted more frequently to verify the implementation of the agreed-upon or guidance.

Once this stage was completed, the group concluded that a tool to guide and streamline nursing consultations is essential, given the overload of activities in daily care. Therefore, the researchers presented a proposed tool to guide nursing consultations, which was extensively discussed and adjusted according to the group’s suggestions. The final model included information regarding social and demographic aspects, general history, family history, past health history, medications currently being used, general physical examination and foot examination, current complaints, nursing diagnosis, and a care plan. The goals established in the care plan were defined jointly with the patient, based on nurses’ history and always considering patients’ health condition and capabilities.

In the fourth workshop, nurses approved the tools and committed to testing them on at least two patients throughout the week. At the next meeting, they discussed the time spent on each consultation, the challenges faced, the strategies used to overcome them, and the adjustments needed to improve the tools and make them more viable.

Finally, at the final workshop, the restructured instrument was distributed, and it was discussed that its use greatly aided patient assessment and facilitated professional-patient interaction. The time to administer/complete all instruments ranged from 15 to 25 minutes. Some considered this time excessively long, while others considered it only slightly longer than that spent in nursing care, since they did not perform systematic nursing consultations.

Based on the nursing diagnoses raised by nurses when applying the instrument during the test nursing consultation, the researchers created a list containing, on the one hand, probable nursing diagnoses and, on the other, possible actions to be guided to patients.

They concluded that as they became more familiar with the tools, time would be reduced. Regarding the feasibility of adopting telenursing, some professionals argued that, due to the high workload at the unit, they would need the collaboration of other team members to ensure telenursing could be effectively implemented at the BHU. Ultimately, it was agreed that each team member should gradually select 30 patients with different risk stratifications to be monitored for a period of one year. The research team was available to answer questions and provide any necessary support during this period.

Overall, the proposal was well received by nurses: the group valued the workshops, felt motivated, and recognized telenursing as a promising strategy for organizing care. Concerning implementation, it was agreed at the end of the workshops that each nurse would gradually select 30 patients from different risk strata for 12-month follow-up. However, it was observed that only three nurses fully complied with the agreement; six followed fewer patients; two did not complete the 12-month follow-up; and five did not even begin follow-up. In summary, engagement can be classified as fair.

Among the factors possibly associated with limited adherence, care overload, staff turnover, lack of time for nursing consultations and care plan development (15-25 minutes per patient), the need for team collaboration, and resource constraints by patients stood out. Greater managerial support and an understanding of nursing’s strategic role in the follow-up of people with chronic conditions could have favored implementation and enhanced results.

## DISCUSSION

The use of educational workshops is a health education strategy that can be used in different contexts and situations, as it is based on collaboration among participants to achieve the goal. Based on the educational workshops held with nurses, challenging aspects of implementing telenursing were identified, as municipalities have different care and work conditions. Collecting information through an online form prior to the workshops helped to better direct the discussions, in addition to identifying the characteristics of care in health units and a more detailed understanding of the weaknesses and strengths experienced by professionals in their daily care of people with HT and DM in PHC.

The content discussed in the educational workshops was defined by the participants themselves, representing what they considered essential for comprehensive care for patients with chronic conditions. By participating in the workshops, nurses were able to understand the positive impact that technology could have on the quality of care provided and patient adherence. A similar result was observed in a study in Spain that assessed the effects of a course on the use of ICTs in PHC, which concluded that telenursing, in addition to monitoring patients with chronic diseases, encourages and helps improve health behaviors and promotes self-efficacy, contributing to strengthening a more effective nurse-patient relationship^(7)^.

The obstacles highlighted by participants in this study included limited time for nursing consultations, insufficient human resources, and inadequate infrastructure. This is not very different from what was found in a study conducted with physicians and nurses, which highlighted the lack of adequate training, difficulties in integrating technologies into routine care, and issues related to system reliability^(8)^. These aspects reflect the need for continued investment and public policies that ensure technical and operational support for the adoption of technologies in PHC.

Another relevant aspect was the importance of raising awareness and engaging professionals in educational workshops, a condition that supports the results of the study with PHC nurses from the 17^th^ HR of Paraná, which considered the implementation of an educational intervention as a fundamental CHE strategy for improving care in PHC^(9)^.

CHE implementation is fraught with challenges related to work overload, lack of planning, devaluation of its actions or misinterpretation of its guidelines by managers, high staff turnover in units, and low adherence to CHE actions in services. However, providing training to professionals working in services directly influences practice, as incorporating the recommendations learned into their daily work improves the quality of patient care^(9)^.

CHE actions, through educational interventions, must be facilitated and inserted into nurses’ daily lives so that their care skills and abilities are strengthened, reflecting in a qualified practice and assertive decisions^(9)^. To this end, collaboration between managers, professionals, and the public is essential, fostering an environment conducive to continuous learning and improvement of care processes. Managers must provide technical, financial, and human support to make these activities feasible. Professionals need to understand their role and actively contribute to the implementation of the model. In turn, the public must be made aware of the importance of self-care in managing chronic conditions^(10)^.

Finally, we suggest that future studies explore patient experiences and outcomes associated with telenursing in different Brazilian contexts to strengthen the evidence base and guide best practices. This will contribute to and favor the effective implementation of technology as a tool for managing chronic conditions and improving the quality of healthcare.

### Study limitations

Potential limitations of this study include the online workshops, the small number of participants, and technical difficulties for some in the group using cameras and microphones, which led some to interact only via chat, limiting oral communication. Furthermore, workload, coupled with a lack of managerial support, discouraged some nurses from participating in the proposed practical activities.

### Contributions to nursing

This study provided relevant contributions to nursing in PHC by demonstrating the viability of telenursing as a complementary strategy in the care of patients with HT and DM. Through educational workshops, nurses developed practical tools for risk stratification to support and systematize nursing consultations and telecontacts, adapted to local constraints, such as the scarcity of laboratory tests. Furthermore, the study highlighted the importance of CHE in training professionals to integrate/insert technologies into routine care, overcoming challenges such as work overload and inadequate infrastructure. The results reinforce the need for public policies that support telehealth, ensuring resources and continuing training so that nursing can offer more agile, personalized, and effective care to chronic patients in PHC.

## FINAL CONSIDERATIONS

The nurses who participated in developing the telenursing proposal identified this strategy as a promising tool for managing chronic conditions, considering that it allows for expanded access and improved monitoring of patients living with these conditions. However, they emphasized the need to systematize the process, in addition to having management support in terms of equipment provision, adequate allocation of human resources, and continuing training. They also emphasized the importance of developing a protocol based on the Chronic Conditions Care Model, already adopted in the state of Paraná, considering the specificities of each context while being feasible for implementation in all municipalities.

For telenursing to be effectively incorporated as a care strategy into the daily practices of PHC services, the protocol must include: criteria for identifying patients eligible for this technology; guidelines for determining the frequency of telenursing; and clinical and psychosocial assessment tools that allow for the establishment of agreed-upon goals. Goals must be compatible with the self-care needs and conditions of each patient.

Despite the limitations, the results obtained are relevant. Although telemonitoring is still an incipient practice in Brazilian literature, the reported experience confirmed the need for auxiliary resources to monitor patients with chronic conditions. Furthermore, the importance of developing specific instruments for this purpose was highlighted, with the active participation of the professionals who will use them in their daily work. It is believed that disseminating the study may spark greater interest in this practice, especially within PHC, contributing to the expansion and improvement of care for patients with chronic conditions in Brazil.

## Data Availability

The research data are available within the article.
