# Supplementary material for: Educational workshops on telenursing in the monitoring of chronic conditions: an experience report
Source: Rev Bras Enferm. 2026 Jul 6;79:e20250246. doi: 10.1590/0034-7167-2025-0246 (PMC13336228; doi:10.1590/0034-7167-2025-0246)
Supplement: Translation

# Translation: Oficinas educativas sobre telenfermagem no acompanhamento de condições crônicas: relato de experiência

**DOI:** 10.1590/0034-7167-2025-0246pt

**Authors:** Iven Giovanna Trindade Lino, Luciana de Alcantara Nogueira, Elen Ferraz Teston, Sonia Silva Marcon

**Affiliations:** I Universidade Estadual de Maringá. Maringá, Paraná, Brasil; II Universidade Federal do Paraná. Curitiba, Paraná, Brasil; III Universidade Federal de Mato Grosso do Sul. Campo Grande, Mato Grosso do Sul, Brasil

**Keywords:** Atenção Primária à, Saúde, Enfermagem, Telenfermagem, Condições Crônicas, Tecnologias da Informação e Comunicação.

## Abstract

**Objetivos:**

relatar a realização de oficinas educativas sobre o uso da telenfermagem no acompanhamento de pessoas com hipertensão arterial e diabetes na Atenção Primária à Saúde.

**Métodos:**

relato de experiência, realizada entre maio e julho de 2021, que envolveu levantamento das características da assistência, discussão sobre possibilidades da telenfermagem, construção de instrumentos e testagem da tecnologia.

**Resultados:**

diagnóstico situacional apontou insatisfação com os resultados da assistência ofertada. Os 16 enfermeiros participantes reconheceram o potencial da telenfermagem, mas apontaram a falta de tempo e insuficiência de recursos humanos e de infraestrutura como possíveis limitações. Participaram da elaboração de formulário para avaliação da aptidão e estratificação de risco do usuário e de roteiro para consulta de enfermagem e plano de cuidados.

**Considerações Finais:**

oficinas educativas são eficazes na capacitação de enfermeiros, confirmando a importância da educação permanente para subsidiar a implementação de mudanças na prática assistencial, como o uso da telenfermagem.

## INTRODUÇÃO

A telenfermagem tem se consolidado como uma estratégia inovadora e eficaz para o acompanhamento de pessoas com doenças crônicas não transmissíveis, especialmente a hipertensão arterial (HA) e o diabetes *mellitus* (DM). Ao integrar tecnologias digitais e comunicação remota, essa modalidade de cuidado permite a ampliação do acesso aos serviços de saúde, redução de complicações e promoção do autocuidado^(1)^.

A Resolução COFEN nº 696/2022, alterada pelas Resoluções nº 707/2022 e nº 717/2023, normatiza a atuação da enfermagem na saúde digital e no uso das tecnologias de informação e comunicação (TICs), estabelecendo que, nas ações mediadas por TICs, é imprescindível o consentimento do usuário envolvido ou do seu responsável legal, sendo passível de desistência a qualquer tempo, com a consequente retirada do consentimento. Na prática, a resolução autoriza os enfermeiros a fazer uso da telenfermagem, isto é, realizar consulta de enfermagem, interconsulta, consultoria, monitoramento, educação em saúde e acolhimento da demanda espontânea mediadas por TICs.

Essa modalidade de cuidado expande o acesso aos serviços de saúde para locais de difícil acesso, reduz o custo e atende às mudanças nos perfis epidemiológicos, como o envelhecimento da população, o aumento das doenças crônicas e os agravos infectocontagiosos. Estima-se que, até 2030, mais da metade dos serviços de saúde será oferecida virtualmente, refletindo a crescente preferência dos pacientes por este modelo de atendimento^(2)^. Além disso, apresenta-se como uma ferramenta essencial de apoio para um relacionamento mais dinâmico e eficaz entre pacientes e profissionais, especialmente no âmbito da Atenção Primária à Saúde (APS)^(3)^.

Estudo realizado com enfermeiros da APS concluiu que a telenfermagem é considerada uma ferramenta útil para o acompanhamento de pessoas com condições crônicas, desde que haja recursos humanos, materiais e capacitações adequadas disponíveis^(4)^. Todavia, cabe destacar que, para incorporação desta modalidade assistencial no cotidiano do trabalho, é fundamental a aceitação dos profissionais e dos usuários, assim como a existência de protocolos e fluxos sistematizados que subsidiem a condução do trabalho, apoiando as ações de saúde e o alcance dos objetivos traçados^(5)^. Ressalta-se que as pesquisas que exploram a experiência dos pacientes e profissionais com o uso da telenfermagem são limitadas e conduzidas majoritariamente em países desenvolvidos, como o Reino Unido e os Estados Unidos da América^(1)^_._

Diante desse cenário, a educação em saúde pode ser uma importante estratégia para qualificar o uso da telenfermagem na APS. Entre as diferentes abordagens da educação em saúde, as oficinas educativas se destacam por constituírem espaço colaborativo para a troca de ideias, permitindo a análise de desafios que exigem mudanças e fortalecem a conexão entre conhecimento e prática, além de estimular o diálogo e a construção coletiva do saber. Em síntese, essa abordagem prepara os profissionais para identificar demandas em saúde e atuar de forma mais eficaz^(6)^.

As oficinas educativas com foco na telenfermagem, portanto, podem sensibilizar e aproximar os profissionais acerca dessa nova possibilidade de cuidado. No entanto, na literatura nacional, não foram identificados estudos que apoiassem os profissionais de enfermagem para o uso da tecnologia em seu cotidiano assistencial, o que motivou a realização deste relato.

## OBJETIVOS

Relatar a realização de oficinas educativas sobre o uso da telenfermagem no acompanhamento de pessoas com HA e diabetes na APS.

## MÉTODOS

Trata-se de relato da experiência vivenciada durante estudo matricial desenvolvido no âmbito da 15ª Regional de Saúde (RS) do estado do Paraná, Brasil, realizado entre maio e junho de 2022, o qual propôs o uso do telemonitoramento como recurso de apoio à autogestão da doença por pessoas com DM e HA.

Os participantes da experiência foram 16 enfermeiros que atuavam em Unidades Básicas de Saúde (UBSs) de municípios integrantes da 15ª RS. Inicialmente, para viabilizar a participação dos enfermeiros, o setor de educação permanente em saúde (EPS) da RS encaminhou um ofício aos coordenadores da APS dos 29 municípios informando sobre a pesquisa e seus objetivos, solicitando que o mesmo fosse encaminhado aos enfermeiros da APS, juntamente com o *link* de acesso a questionário no *Google Form*s^®^, o qual deveria ser respondido no prazo de 15 dias. Transcorridos 30 dias e diante da baixa devolutiva, um novo ofício foi encaminhado aos coordenadores reforçando a importância da colaboração na pesquisa.

O questionário foi elaborado pelas autoras, e consistiu-se em quatro partes: I) Caracterização sociodemográfica: sexo, idade, tempo de formação e especialização na área da saúde da família ou saúde pública; II) Características da assistência às pessoas com HA e/ou DM, abordando o acompanhamento realizado e percepções sobre as facilidades e dificuldades desse acompanhamento; III) Conhecimento e percepções sobre tecnologias de informação e comunicação e capacitações anteriores; IV) Possibilidade do uso da telenfermagem no cotidiano: disposição para utilizar e disponibilidade de recursos físicos, estruturais e humanos na unidade de saúde, além de interesse em participar de oficinas para o uso dessa tecnologia. Incluía o Termo de Consentimento Livre e Esclarecido, sendo o seu aceite uma condição necessária para acessar o formulário de pesquisa.

Os enfermeiros que sinalizaram interesse em participar das oficinas foram contatados e informados sobre como e quando seriam realizadas. Confirmado o interesse, todos foram inseridos em um grupo no aplicativo de mensagens *WhatsApp* ^®^ para facilitar a comunicação, o compartilhamento de informações sobre a condução das oficinas e fortalecer o vínculo com os pesquisadores. O primeiro encontro foi agendado de acordo com a disponibilidade da maioria.

As oficinas, um total de cinco, foram semanais e realizadas em horário e dia definidos pela maioria como mais apropriados (quarta-feira às 15 horas). Todas as oficinas foram videogravadas após autorização dos participantes, e tiveram duração média de 60 minutos. No dia anterior a cada encontro, a pesquisadora enviava um lembrete no grupo do *WhatsApp* ^®^ reforçando a importância da presença de todos e o cumprimento da tarefa da semana anterior. As oficinas foram conduzidas por uma das autoras, enfermeira, doutoranda em enfermagem com experiência na coleta e análise de dados qualitativos e que não possuía nenhum vínculo com os participantes.

Para condução e realização das oficinas educativas, após debate e acordo entre os participantes, foi elaborado um plano de atividades a partir das respostas do questionário *online*, o qual foi organizado em cinco etapas, como se pode ver na Figura 1.

**Figura 1:** Fluxograma explicativo dos momentos dos encontros

O desenvolvimento desta pesquisa seguiu todos os preceitos éticos disciplinados pelas Resoluções n^o^ 466/2012 e n^o^ 510/2016 do Conselho Nacional de Saúde, e as orientações para procedimentos em pesquisas com qualquer etapa em ambiente virtual - Comissão Nacional de Ética em Pesquisa/2021. O projeto foi submetido à apreciação e aprovado pelo Comitê de Ética em Pesquisa.

## RESULTADOS

Dos 289 enfermeiros atuantes na 15ª RS do Paraná, 65 responderam ao questionário; 42 manifestaram interesse em participar das oficinas educativas; e 16 participaram efetivamente.

Na primeira oficina, o grupo discutiu as informações obtidas com a aplicação do questionário *online* e refletiram a realidade vivenciada por cada um do grupo, destacando as fragilidades e potencialidades percebidas na assistência às pessoas com HA e/ou DM na UBS onde atuam. O objetivo dessa atividade foi conhecer os participantes, compreender seu contexto de trabalho e identificar como era prestada a assistência às pessoas com HA e/ou DM.

Na segunda e terceira oficinas, foram discutidos os princípios, os limites, as possibilidades e os recursos necessários para o uso da telenfermagem na APS. Durante as discussões, surgiu a preocupação sobre quais usuários estariam aptos a receber este tipo de assistência e os aspectos a serem considerados, tais como frequência de procura pela UBS, vulnerabilidade social e habilidade para lidar com dispositivos móveis, pois o usuário precisaria ter telefone e habilidade para utilizar a tecnologia de forma independente.

Durante esses dois encontros, o grupo concluiu que a telenfermagem seria uma estratégia promissora e com potencial para contribuir com a organização da assistência, definindo as etapas necessárias para sua operacionalização. São elas: 1) Identificar os usuários com HA e/ou DM aptos para receber telecontatos; 2) Utilizar a estratificação de risco na definição da periodicidade dos telecontatos; 3) Realizar consulta de enfermagem presencial para o levantamento de problemas, elaboração do plano de cuidados e definição conjunta das metas a serem pactuadas; 4) Realizar telecontatos para acompanhar o cumprimento das metas pactuadas e verificar as facilidades e dificuldades enfrentadas pelo usuário no gerenciamento de sua condição de saúde; e 5) Estabelecer novas metas ou retomar a meta inicial. Todavia, para inseri-la na rotina de trabalho, seria necessário testar sua viabilidade e existir instrumentos específicos para nortear as ações.

O grupo listou aspectos importantes para constarem nos instrumentos, e destacou a necessidade de considerar as características e especificidades dos usuários que seriam assistidos e as distintas realidades dos municípios, para que os instrumentos fossem úteis e possíveis de serem utilizados por todos os participantes. O primeiro instrumento elaborado abordou a aptidão do usuário para a telenfermagem e a estratificação de risco para definir a periodicidade das ligações. Destaca-se que, embora a Linha Guia da Hipertensão e da Diabetes no Estado do Paraná apresente critérios para identificação do estrato de risco dos pacientes, alguns enfermeiros relataram que seus municípios não tinham cota para a realização de alguns exames laboratoriais necessários para aquela classificação. Por esta razão, esses exames não eram realizados, muito menos com a periodicidade necessária, ou só era quando o próprio paciente conseguir custear.

Para agilizar o processo, a partir das sugestões do grupo, as pesquisadoras elaboraram um esboço de instrumento para ser aprimorado pelo grupo, o qual foi enviado a todos durante a semana, para que as discussões na oficina fossem mais dinâmicas. Para que os critérios propostos pudessem ser adotados por todos os municípios, optou-se por fazer uma adaptação dos critérios da Linha Guia do Paraná, com atribuição de pontos para algumas condições/situações. Desse modo, na verificação da aptidão do usuário, foram consideradas posse de telefone fixo ou móvel, habilidade para manusear o aparelho telefônico e receber ligações, pessoas com quem reside, presença de dificuldade auditiva e/ou visual, e se gostariam de ser acompanhadas pela telenfermagem. Para estratificar o risco, elencaram-se as seguintes variáveis: idade; Índice de Massa Corporal; circunferência abdominal; tabagismo; procura de unidade de pronto atendimento e/ou internação nos últimos 12 meses por descontrole pressórico ou glicêmico; cirurgia; complicações; uso de medicamentos de rotina; e os parâmetros pressóricos e glicêmicos. Assim, tomando como base os parâmetros estabelecidos na Linha Guia do Paraná, consideraram-se como risco baixo, moderado e alto os pacientes que apresentam as variáveis elencadas, conforme ilustrado no Quadro 1.

**Quadro 1:** Critérios para estratificação de risco definidos por enfermeiros com base na Linha Guia do Paraná, Maringá, Paraná, Brasil, 2025

Condição crônica	Variáveis	Risco
DM	Diagnóstico de DM; glicemia até 130 mg/dL; usa um tipo de medicação; segue dieta e/ou pratica atividade física; não fuma; não ingere bebida alcoólica; glicemia pós-prandial < 180 mg/dL; ausência de complicações de DM2; procurou unidade de saúde nos últimos 12 meses por descontrole glicêmico.	Baixo
	Usa dois ou mais medicamentos antidiabéticos; não segue dieta e/ou não pratica atividade física; ingere bebida alcoólica e/ou fuma; glicemia pós-prandial ≥ 180 mg/dL; ausência de complicações de DM2; necessidade de procurar serviços de saúde de uma a duas vezes nos últimos 12 meses por descontrole glicêmico.	Médio
	DM1 ou DM2; usa insulina; complicações (macro ou microvascular); procurou serviços de saúde ≥ três vezes em um ano por descompensação glicêmica.	Alto
HA	Diagnóstico de HA; uso regular de medicamento; pressão sistólica de 130-139 mmHg ou diastólica de 85-89 mmHg; não procurou os serviços de saúde nos últimos 12 meses por descontrole pressórico.	Baixo
	Pressão arterial ≤ 130/80 mmHg; usa de dois a três anti-hipertensivos; segue dieta e/ou pratica atividade física; fuma e/ou bebe; procurou os serviços de saúde de uma a duas vezes no ano por descontrole pressórico.	Médio
	Usa de dois a três medicamentos; pressão arterial ≥ 180 mmHg ou diastólica ≥ 110 mmHg; não segue dieta e/ou atividade física; fuma e/ou bebe; apresenta complicações cardiovasculares. procurou os serviços de saúde mais de três vezes em 12 meses por descontrole pressórico.	Alto

*HA - hipertensão arterial; DM - diabetes mellitus.*

*A priori*, o grupo definiu que a estratificação de risco definiria a periodicidade das ligações, sendo que, para usuários com HA e/ou DM de baixo e médio risco, cuja pactuação das metas tem como propósito a mudança de comportamentos de risco, as ligações deveriam ocorrer a cada três meses, totalizando quatro ligações no período de 12 meses. Já para os usuários estratificados como de alto risco, cujo enfoque da pactuação é concentrado no manejo/gestão de caso, as ligações deveriam ser mensais.

No entanto, ponderou-se que a periodicidade estabelecida poderia variar de acordo com as metas pactuadas nos planos de cuidados e com a evolução da condição de saúde/comportamento do usuário. Em outras palavras, na consulta de enfermagem presencial, o enfermeiro poderia se deparar com um usuário com DM1 que, de acordo com os critérios estabelecidos, seria estratificado como de alto risco, mas, ao constatar que ele tem hábitos saudáveis, autocuidado satisfatório com índices glicêmicos dentro dos padrões normais, os telecontatos não precisariam ser mensais, mas sim trimestrais.

Por outro lado, usuários estratificados como sendo de baixo ou médio risco poderiam apresentar comportamento em saúde bastante prejudiciais, com muitos hábitos de vida não saudáveis. Nesses casos, as metas a serem alcançadas precisam ser pactuadas aos poucos, e algumas mais emergentes precisam ser monitoradas com maior frequência e de forma mais incisiva. Desse modo, seria necessário que o usuário fosse contatado com maior frequência para verificar a implementação do que foi pactuado ou orientado.

Superada essa etapa, o grupo concluiu sobre a importância de instrumento para direcionar e agilizar a consulta de enfermagem, visto que há sobrecarga de atividades no cotidiano da assistência. Com isso, os pesquisadores apresentaram uma proposta de instrumento para guiar a consulta de enfermagem, que foi amplamente debatida e ajustada conforme sugestões do grupo. O modelo final inclui informações referentes a aspectos sociais e demográficos, anamnese geral, histórico familiar, história de saúde pregressa, medicações em uso, exame físico geral e dos pés, queixas atuais, diagnóstico de enfermagem, e plano de cuidados. As metas estabelecidas no plano de cuidados eram definidas em conjunto com o usuário, com base na anamnese realizada pelo enfermeiro e sempre considerando a condição de saúde e as possibilidades do usuário.

Na quarta oficina, os enfermeiros aprovaram os instrumentos e se comprometeram a testá-los em pelo menos dois usuários ao longo da semana. No encontro seguinte, foram discutidos o tempo gasto em cada consulta, as dificuldades enfrentadas, as estratégias utilizadas para superá-las, e os ajustes necessários para aprimorar os instrumentos e torná-los mais viáveis.

Por fim, na última oficina, o instrumento reestruturado foi enviado, sendo discutido que a utilização deles auxiliou sobremaneira na avaliação do usuário, facilitando, inclusive, a interação profissional-usuário. O tempo para aplicação/preenchimento de todos os instrumentos variou entre 15 e 25 minutos. Alguns consideraram esse tempo demasiadamente longo, enquanto outros ponderaram que o tempo foi só um pouco mais longo do que aquele dispensado no atendimento de enfermagem, visto que não realizavam a consulta de enfermagem sistematizada.

Com base nos diagnósticos de enfermagem levantados pelos enfermeiros na aplicação do instrumento durante a consulta de enfermagem teste, as pesquisadoras elaboraram uma relação contendo, de um lado, prováveis diagnósticos de enfermagem e, de outro, possíveis ações a serem orientadas aos usuários.

Concluíram que à medida que se familiarizassem com os instrumentos, o tempo seria reduzido. Referente à viabilidade de adotar a telenfermagem, alguns profissionais alegaram que, por conta da elevada demanda de trabalho na unidade, precisariam da colaboração de outros membros da equipe para que a telenfermagem pudesse ser realmente adotada na UBS. Ao final, foi acordado que cada um deveria selecionar, gradativamente, 30 usuários com diferentes classificações de risco para serem acompanhados pelo período de um ano. A equipe de pesquisa se colocou à disposição para esclarecer dúvidas e apoio que fosse necessário durante o período.

De modo geral, a proposta foi bem recebida pelos enfermeiros: o grupo valorizou as oficinas, sentiu-se motivado e reconheceu a telenfermagem como estratégia promissora para organizar a assistência. No que tange à implementação, pactuou-se ao final das oficinas que cada enfermeiro selecionaria, gradativamente, 30 usuários de diferentes estratos de risco para acompanhamento por 12 meses. Observou-se, contudo, que apenas três enfermeiros cumpriram integralmente o acordado; seis acompanharam número inferior de pacientes; dois não realizaram o acompanhamento por 12 meses; e cinco sequer iniciaram o acompanhamento. Em síntese, o engajamento pode ser classificado como regular.

Entre os fatores possivelmente associados à limitação da adesão, destacaram-se a sobrecarga assistencial, a rotatividade de profissionais, a indisponibilidade de tempo para a realização da consulta de enfermagem e elaboração do plano de cuidados (15-25 minutos por usuário), a necessidade de colaboração da equipe, e as restrições de recursos por parte dos usuários. Considera-se que maior apoio gerencial e compreensão do papel estratégico da enfermagem no seguimento de pessoas com condições crônicas poderiam ter favorecido a implementação e potencializado os resultados.

## DISCUSSÃO

A utilização de oficinas educativas é uma estratégia de educação em saúde que pode ser utilizada em diferentes contextos e situações, pois tem como premissa a colaboração entre os participantes para o cumprimento da meta. A partir das oficinas educativas realizadas com enfermeiros, perceberam-se aspectos desafiadores à implementação da telenfermagem, pois os municípios apresentam diferentes condições de assistência e trabalho. A coleta de informações por formulário *online*, antes das oficinas, contribuiu para melhor direcionar as discussões, além de identificar as características da assistência nas unidades de saúde e uma compreensão mais detalhada das fragilidades e potencialidades experenciadas pelos profissionais no cotidiano na assistência às pessoas com HA e DM na APS.

Os conteúdos discutidos nas oficinas educativas foram definidos pelos próprios participantes, representando, portanto, o que eles consideravam essencial para uma assistência integral aos usuários com condições crônicas. Ao participarem das oficinas, os enfermeiros puderam compreender o impacto positivo que a tecnologia poderia ter na qualidade da assistência ofertada e na adesão aos cuidados pelos pacientes. Resultado semelhante foi observado em estudo na Espanha que avaliou os efeitos de um curso sobre o uso das TICs na APS, o qual concluiu que a telenfermagem, além de acompanhar pacientes com doenças crônicas, incentiva e auxilia na melhoria dos comportamentos de saúde e na promoção da autoeficácia, contribuindo para o fortalecimento de um relacionamento mais eficaz entre enfermeiro e paciente^(7)^.

Os obstáculos apontados pelos participantes do presente estudo incluíram limitação de tempo para consultas de enfermagem, recursos humanos insuficientes e infraestrutura inadequada, não diferindo muito do encontrado em estudo realizado com médicos e enfermeiros, os quais apontaram a falta de treinamento adequado, dificuldades em integrar as tecnologias na rotina e questões relacionadas à confiabilidade dos sistemas^(8)^. Esses aspectos refletem a necessidade de investimentos contínuos e políticas públicas que garantam suporte técnico e operacional para a adoção de tecnologias na APS.

Outro aspecto relevante foi a importância da sensibilização e do engajamento dos profissionais nas oficinas educativas, condição que corrobora o resultado do estudo com enfermeiros da APS da 17ª RS do Paraná, o qual considerou a realização de uma intervenção educativa como estratégia fundamental de EPS para o aprimoramento da assistência na APS^(9)^.

A efetivação da EPS é permeada por desafios relacionados à sobrecarga de trabalho, falta de planejamento, desvalorização de suas ações ou descaracterização de suas diretrizes pelos gestores, rotatividade de trabalhadores nas unidades, e baixa adesão a ações de EPS nos serviços. No entanto, a realização de capacitação para os profissionais inseridos nos serviços exerce uma influência direta na prática, pois, ao incorporarem no dia a dia profissional as recomendações aprendidas, aprimoram a qualidade do cuidado prestado aos pacientes^(9)^.

Ações de EPS, por meio de intervenções educativas, devem ser facilitadas e inseridas no cotidiano dos enfermeiros, para que suas competências e habilidades assistenciais sejam fortalecidas, refletindo em uma prática qualificada e com decisões assertivas^(9)^. Para tanto, a colaboração entre gestores, profissionais e a população é essencial, favorecendo um ambiente propício à aprendizagem contínua e à melhoria dos processos de cuidado. Gestores devem fornecer suporte técnico, financeiro e humano para viabilizar as atividades. Os profissionais precisam compreender seu papel e contribuir ativamente para a implementação do modelo. Por sua vez, a população deve ser sensibilizada sobre a importância do autocuidado no manejo das condições crônicas^(10)^.

Por fim, sugere-se que estudos futuros explorem as experiências dos pacientes e os desfechos associados à telenfermagem em diferentes contextos brasileiros, de modo a fortalecer a base de evidências e orientar as melhores práticas. Isso contribuirá e favorecerá a implementação eficaz da tecnologia como ferramenta para o manejo das condições crônicas e a melhoria da qualidade do cuidado em saúde.

### Limitações do estudo

Como possíveis limitações do estudo, destacam-se a realização das oficinas em formato *online*, o número reduzido de participantes e dificuldades técnicas de parte do grupo para utilizar câmera e microfone, o que levou alguns a interagir apenas pelo *chat*, restringindo manifestações orais. Ademais, a sobrecarga de trabalho, associada à ausência de apoio gerencial, desestimulou a participação de alguns enfermeiros na execução das atividades práticas propostas.

### Contribuições para a área da enfermagem

Este estudo trouxe contribuições relevantes para a enfermagem na APS, ao demonstrar a viabilidade da telenfermagem como estratégia complementar no cuidado a pacientes com HA e DM. Por meio de oficinas educativas, os enfermeiros desenvolveram instrumentos práticos para a estratificação de risco, para subsidiar e sistematizar a consulta de enfermagem e os telecontatos, adaptados às limitações locais, a exemplo da escassez de exames laboratoriais. Além disso, o estudo evidenciou a importância da EPS na capacitação de profissionais para integrar/inserir tecnologias na rotina assistencial, superando desafios como sobrecarga de trabalho e infraestrutura inadequada. Os resultados reforçam a necessidade de políticas públicas que apoiem a telessaúde, garantindo recursos e treinamentos contínuos para que a enfermagem possa ofertar um cuidado mais ágil, personalizado e eficaz aos usuários crônicos na APS.

## CONSIDERAÇÕES FINAIS

Os enfermeiros que participaram da elaboração da proposta de telenfermagem identificaram essa estratégia como ferramenta promissora para o gerenciamento de condições crônicas, considerando que ela permite ampliar o acesso e melhorar o monitoramento dos usuários que convivem com essas condições. No entanto, ressaltaram a necessidade de sistematizar o processo, além de contar com o apoio da gestão em termos de fornecimento de equipamentos, alocação adequada de recursos humanos e capacitação contínua. Enfatizaram, também, a importância de desenvolver um protocolo fundamentado no Modelo de Atenção às Condições Crônicas, já adotado no estado do Paraná, considerando as especificidades de cada contexto e, ao mesmo tempo, sendo viável para implementação em todos os municípios.

Para que a telenfermagem seja efetivamente incorporada como uma estratégia assistencial nas práticas diárias dos serviços de APS, o protocolo deve incluir: critérios para identificar os usuários aptos a utilizarem essa tecnologia; diretrizes para definir a frequência dos telecontatos; e instrumentos de avaliação clínica e psicossocial que permitam o estabelecimento de metas pactuadas. As metas devem ser compatíveis com as necessidades de autocuidado e as condições de cada usuário.

A despeito das limitações, os resultados obtidos são relevantes, pois embora o telemonitoramento ainda seja uma prática incipiente na literatura brasileira, a experiência relatada confirmou a necessidade de utilizar recursos auxiliares para o acompanhamento de usuários com condições crônicas. Além disso, destacou-se a importância de desenvolver instrumentos específicos para essa finalidade, com a participação ativa dos profissionais que irão utilizá-los no cotidiano de trabalho. Acredita-se que a divulgação do estudo possa despertar maior interesse por essa prática, especialmente no âmbito da APS, contribuindo para a ampliação e qualificação do cuidado aos pacientes com condições crônicas no Brasil.

## Data Availability

Os dados de pesquisa estão disponíveis no corpo do artigo.
